# Organoids of epithelial ovarian cancer as an emerging preclinical in vitro tool: a review

**DOI:** 10.1186/s13048-019-0577-2

**Published:** 2019-11-08

**Authors:** Sander Dumont, Ziga Jan, Ruben Heremans, Toon Van Gorp, Ignace Vergote, Dirk Timmerman

**Affiliations:** 1 Division of Gynecologic Oncology, Department of Gynecology and Obstetrics, University Hospitals Leuven, Herestraat 49, 3000 Leuven, European Union Belgium; 2 Department of Gynecology, Klinikum Klagenfurt, Klagenfurt, European Union Austria; 3 Department of Development and Regeneration, KU Leuven, Leuven, European Union Belgium; 4 Department of Oncology, Leuven Cancer Institute, KU Leuven, Leuven, European Union Belgium

**Keywords:** Cell culture, Genetics, Stem cells, Therapy, Diagnostics, Drug screening

## Abstract

Epithelial ovarian cancer (EOC) remains the most lethal gynecological cancer in developed countries, indicating the need for further research. Although current cancer models prove useful, they have major limitations. Organoids, a novel in vitro 3D cell culture technique, derived from stem cells, could provide a bridge between the current preclinical platforms. However, this technique is still in its early stages. After conducting a systematic literature search, only sixteen manuscripts concerning ovarian related organoids could be retrieved.

In this review, we discuss current tumor models, including organoids and provide a comprehensive review about organoids of ovarian tissue. Potential future applications are addressed, proving organoids to be an interesting platform for modeling tumorigenesis, drug testing and screening and other applications. Recent advancements could usher in a new era of highly personalized medicine in EOC.

## Introduction

Epithelial ovarian cancer (EOC) is the most lethal gynecological cancer and the fifth largest cause of cancer-related death in women in Europe [1]. The unfavorable prognosis of this disease is due to a combination of late diagnosis, mostly at an advanced stage, and frequent relapses with increasing resistance to chemotherapy and targeted therapy.

Organoids, a novel in vitro 3D cell culture derived from stem cells of various organs and tissues, could provide an interesting in vitro preclinical platform to address these issues and could be the next step for personalized medicine. In this paper, we aim to give a general overview of the current knowledge about this promising tool and discuss possible future clinical applications in EOC.

**Fig. 1:**
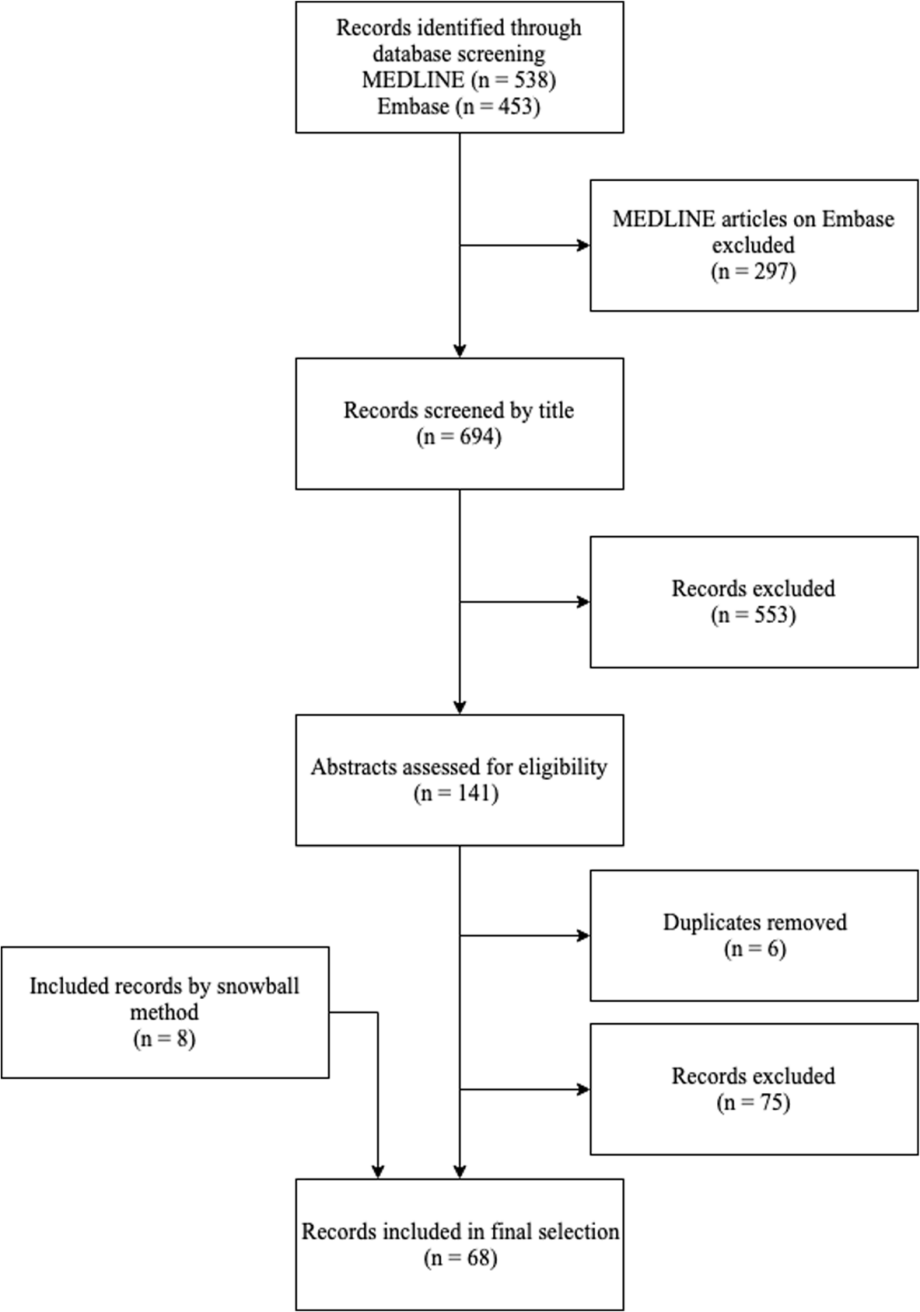
Flowchart depicting the selection methods for relevant manuscripts about organoids

We performed a dual search: First, we searched for literature on current insights and general knowledge about organoids. We systematically searched articles January 1st, 2007 until January 14th, 2019 in MEDLINE and Embase using the terms provided in Additional file 1: Table S1. A summary of the search process is provided in Fig. 1.

**Fig. 2:**
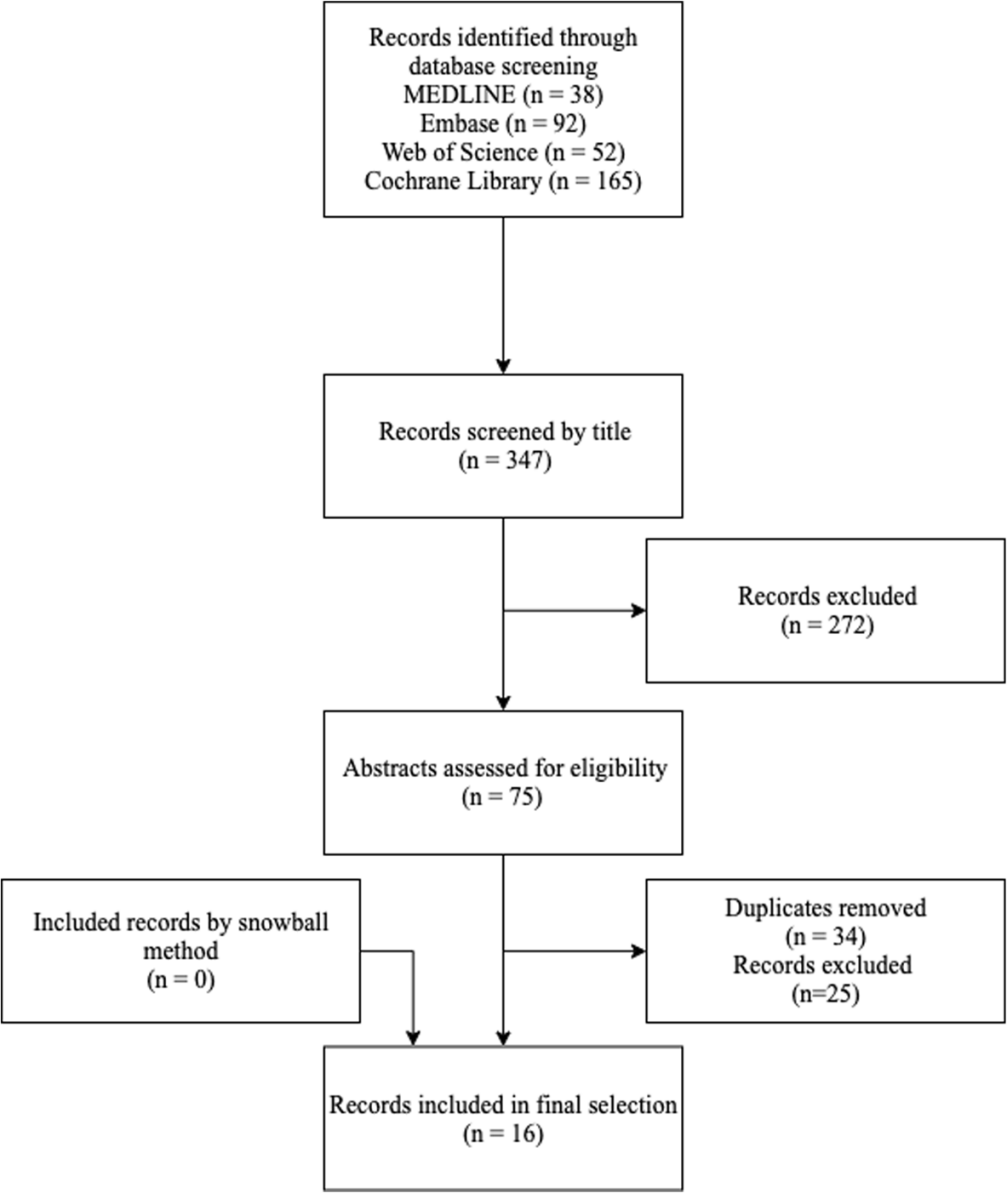
Flowchart depicting the search process for articles about organoids of ovarian or fallopian origin

Second, MEDLINE, Embase, Web of Science and the Cochrane library were searched between January 1st, 2007 until September 29th, 2019, using the terms provided in Additional file 1: Table S2. The search process is depicted in Fig. 2. A total listing of the sixteen retrieved articles can be found in Additional file 1: Table S3. Only nine articles describe organoids of ovarian epithelial cancer [2–10].

## Current tumor models

**Fig. 3:**
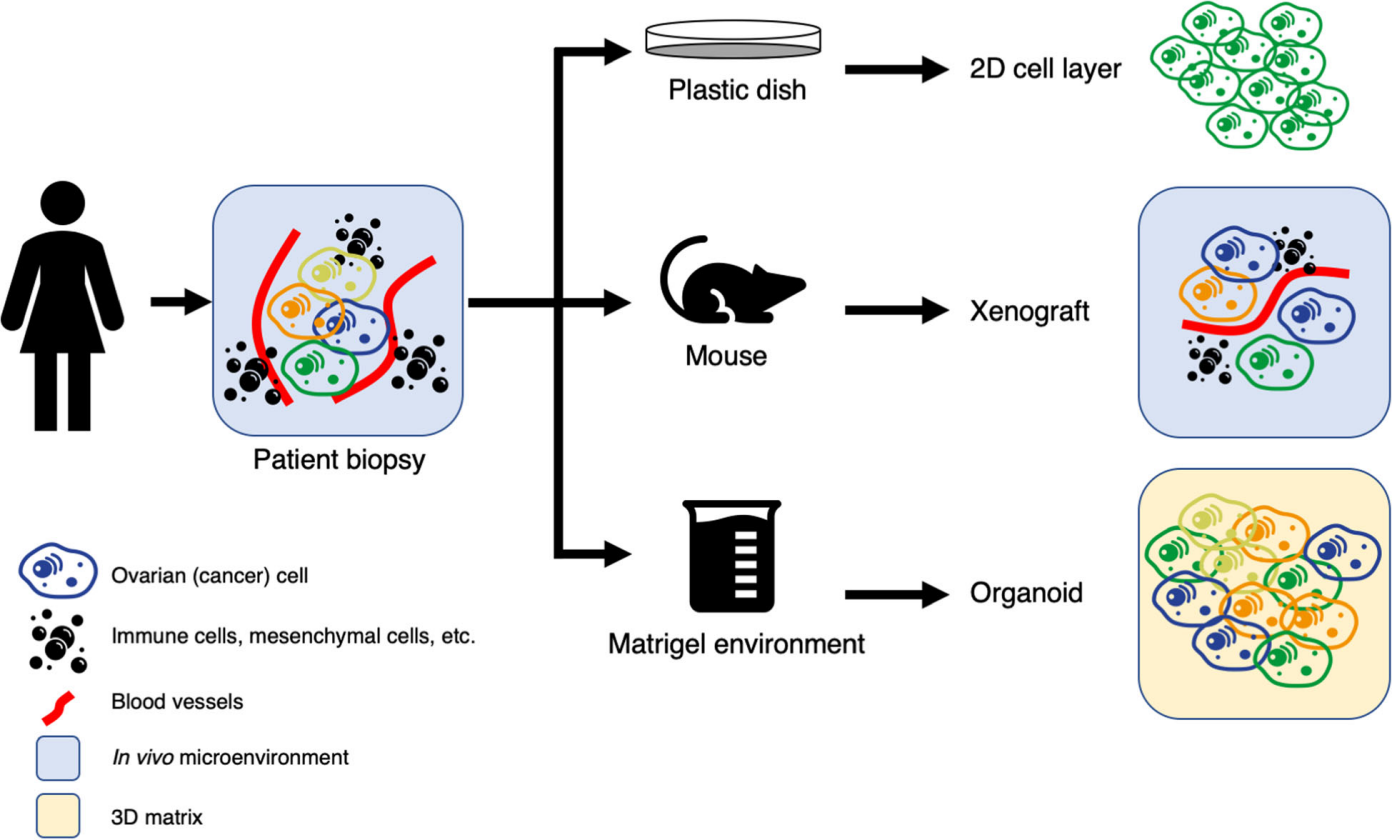
Current techniques for generating a tumor model

Currently, three major tumor models are available [11]. We summarize these techniques in Fig. 3 and provide an overview of advantages and disadvantages in Table 1.

**Table 1:** Overview of cancer cell culture techniques, adapted from [11–13]

Technique	Advantages	Disadvantages
Traditional cell culture (monolayer and spheroids)	- Easily maintained and expanded	- Cancer cells lose grow potential in vitro
	- Genetic manipulation	- Often only one clone expands
	- High-throughput drug screening	- Lower biological stability
	- Most favorable cost-benefit	- Inability to represent the clinical cancer spectrum
Xenografts	- In vivo culture	- Difficult genetic manipulation
	- High heterogeneity possible	- Graft failure
	- Complete microenvironment	- Time and resource consuming
		- Lack of high-throughput drug screening
Organoids	- Long term expansion	- Unknown capability to capturing whole spectrum of in vivo heterogeneity
	- Mimicking in vivo conditions	- Time consuming initiating a model
	- High-throughput drug screening	- No microenvironment
	- Tissue subtype modeling	

The conventional 2D cell culture has long been established and allows for a rapid and reliable growth of cancer cells. However, the major drawback of this technique, is its inability to faithfully reproduce the clinical cancer spectrum [11, 12]. Spheroids are 3D floating cell aggregates in serum-free media, originating from conventional cancer cell lines [8]. These well-rounded sphere-like structures are also unable to fully replicate in vivo nature of tumors, limiting the clinical applicability [12, 14].

In order to create an in vivo environment to cultivate cancer cells, tumor-derived xenografts were created. By transplanting human cancer cells into immunodeficient mice, a more faithful environment for the tumor cells is created, allowing for a more biological stable tumor model with conserved tumor heterogeneity [11]. Although this is a good tumor model, it is not without its drawbacks. A major concern is the time- and resource-demanding nature of this platform, making it unable to perform high throughput drug screens [11].

We further discuss the third model for tumor culture, called organoids. This recent technique creates a bridge between the ease of conventional cell models and applicability of the in vivo xenografts. Additionally, these organoids are less resource-intensive compared to xenografts [13].

## Organoids

### Definition

Organoids can be defined as 3D structures grown from stem cells and consisting of organ-specific cell types that self-organizes through cell sorting and spatially restricted lineage commitment [15]. Following this definition, the first organoids following this definition were described in 2009 by reproducing the stem cell niche from small-intestinal crypts [16]. However, there is no general consensus about this definition [12, 17]. Furthermore, often the term organoid is misused for spheroids and short-term cell cultures [14].

### Origin of organoids

Organoids can be derived from either adult stem cells (ASC) or from pluripotent embryonic stem cells (ESC) and their synthetically induced counterparts, i.e. induced pluripotent stem cells (iPSC) [15]. This important point indicates the major difference between spheroids and organoids; organoids are derived from stem cells, whereas spheroids are not [14, 15]. This implicates organoids have a larger potential to fully replicate the genomic background of the original tissue and are able to form an in vivo-like spatial architecture [12].

These stem cells have the (theoretical) ability to replicate infinitely. With the correct concentrations of growth factors in order to mimic stem cell niche, these stem cells develop into organized tissues with various types of epithelial cells and with the same micro-architecture as the original tissues, healthy or diseased [18]. The required growth factors primarily drive the Wnt-pathway, an essential signal for maintaining the stem cell compartment [19]. Organoids already exist derived from various different organs and tissues (Table 2). Living organoid biobanks have already been established of breast, colorectal, lung, pancreatic and prostate cancer [13, 22]. While certain cells develop more readily into organoids, the exact properties of these organoid-initiating cells are currently unknown [23]. By using gene-editing techniques, it could be possible to further characterize these organoid-initiating properties, aiding in using organoids in other tissue types.

**Table 2:** An overview of established organoids from various human tissues and organs, adapted from [15, 17, 20, 21]

Tissue/Organ	Derived from adult stem cells	Derived from pluripotent stem cells
Adenohypophysis		+
Bile duct		+
Brain		+
Colon	+	
Esophagus	+	
Fallopian tube	+	
Gallbladder	+	
Inner ear		+
Kidney		+
Liver	+	+
Lung	+	+
Mammary gland	+	+
Pancreas	+	+
Prostate	+	
Retina/Optic cup		+
Salivary gland	+	
Small intestine	+	+
Stomach	+	+
Taste buds	+	
Thyroid		+
Ovary	+	+

### Tumor-derived organoids

As stated before, the existing preclinical models prove to be relevant, however with certain limitations when researching cancer. The major disadvantage of these technologies is failing to implement the findings in a clinical setting and thus reducing the efficacy in the discovery of new drugs [22]. These shortcomings could be bypassed by patient-specific cancer organoids that faithfully recapitulate the biological properties of their tumor of origin.

Tumor-derived organoids are cultivated from a resected human tumor biopsy. After processing, iPSCs or ASCs are retrieved from the biopsy, allowing for the generation of organoids [12, 13]. An interesting aspect of tumor-derived organoids is their ability to preserve tumor heterogeneity, as observed in vivo, even in long-term expansion, a major advantage over traditional cell lines [13, 22].

### Relevant applications

#### Gene editing

Using organoids, it would be theoretically feasible to introduce a nearly infinite amount of mutations in the genome itself, allowing for new genetic discoveries and insights [13, 24, 25]. Using the CRISPR/Cas9 technique, the genome of organoids can be relatively easily manipulated with high precision by introducing a double stand break at any location in the genome and insert or delete nucleotides or entire gene sequences [24, 25]. By doing so, gene expression, epigenetic changes or RNA behavior can be altered [25]. This technique has already been used in organoids derived from adult stem cells and its iPSC counterparts.

Monogenetic diseases can be modified, with potential clinical use [25, 26]. Mutations can be introduced or altered, modeling the tumorigenesis and potentially identifying and modifying driver genes in cancer organoids [13, 25]. Reporter cell lines (such as fluorescent proteins) could be introduced, allowing for quick identification of optimal conditions of organoids or as markers for genetic alterations [24, 25]. Lastly, CRISPR/Cas9 could be used in genome-wide screening, allowing to replicate the in vivo genetic changes [25].

#### Modeling disease and development

Organoids provide an insight into embryonal and stem cell development and thus enabling detailed monitoring of each developmental step [18]. A major advantage of these human organoids over animal models is the ability to faithfully mimic human development. A striking example are organoids mimicking human brains, providing a unique insight into human neural growth [18]. Organoids could also be a good model to study the development of cancer. In a bottom-up fashion, the organoids are artificially (by CRISPR/Cas9) modified by a gain-of-function or a loss-of-function mutation, and thus inducing malignancy [27]. The major advantage of this bottom-up approach, over the top-down approach of traditional cell lines, is reducing unnecessary genetic background information. Furthermore, multistep carcinogenesis can be precisely studied using organoids [27].

#### Drug testing and screening

Organoids could be used in order to test or screen drugs and their toxicities in certain pathologies or tissues [24]. The ultimate goals of these organoids would be to provide a preclinical platform for personalized medicine in oncological patients by correlating drug response to genomic, transcriptomic and proteomic alterations. This would reduce the burden of therapy on the patient and could potentially reduce cancer treatment costs [22]. Organoids could also be used for drug testing, streamlining efforts of drug companies for finding new cancer therapies [13, 28]. Furthermore, large biobanks of organoids would allow already developed drugs to be tested ex vivo, providing insight into possible resistance mechanisms [13]. Developing biobanks of organoids could allow for high throughput drug, predicting patient response before administering the therapy [12]. This has already been observed in a proof-of-concept study [29]. By using these biobanks, the ideal patient population for a particular drug could also be selected prior to the clinical trial [22]. By combining drug screening, cancer gene sequencing and biobanking, organoids could prove to be the key to true personalized cancer medicine [12, 13]. However, not all human drugs can be tested on organoids, since these lack angiogenic factors, immune cells or complete niche paracrine signaling [18]. Although, these limitations are being actively researched, different tumor-derived organoids from various organs and for multiple applications are already been established, as summarized in Table 3.

**Table 3:** Tumor-derived organoids and its applications, adapted from [12, 13, 26, 30]

Cancer type	Aim of study
Breast	- Elucidate pathways of ﻿tumorigenesis and metastasis
	- Detect drug response of organoids
	- Validation of disease-causing genomic variations
	- Organoid biobank
Colorectal	- Genetic diversity of patient- derived tumor organoids and the original tumor biopsy
	- Organoid biobank
	- Personalized medicine
	- Modeling specific subtype of colon cancer
Endometrium	- Organoid biobank
	- Precision medicine
Glioblastoma	- Organoid model of non-epithelial tumor
Liver	- Organoid biobank
	- Detection of driver mutations
Pancreatic	- Pancreatic ductal adenocarcinoma modeling and drug screening
	- Organoid model
Prostate	- Tumor modeling
	- Lineage and cell transition monitoring
Renal	Method for renal carcinoma 3D culture
Stomach and esophageal	- Genomic-based classification of gastric cancer
	- New driver mutation detection
	- Personalized medicine
	- Organoid biobank
	- Long-term 3D cultures of human gastric stem cells and bacterial infection study

#### Additional applications

CRISPR/Cas9 could be used to correct germline mutations in patients, such as in cystic fibrosis [18, 25]. The mutated CFTR gene, involved in cystic fibrosis, has already been successfully corrected in patient-derived intestinal organoids [24]. Although controversial, potentially, these organoids could be transplanted into the affected individuals in order to correct the mutation in vivo [13, 18, 24]. Organoids could also be used for transplantation and tissue regeneration purposes [18, 29]. Using iPSC, it could be possible to generate healthy HLA-matched tissues.

### Limitations and challenges

#### Matrigel

Matrigel (Corning & BD Biosciences) is a substitute for an extracellular matrix, derived from mouse sarcoma cells. This is a stiff extracellular matrix, impairing drug penetration to the target tissues and thus potentially reducing the response of the cells compared to an in vivo setting [18, 22]. The non-human nature makes it unsuitable for human subjects due to potential immune reactions or other potential side effects, causing a major limitation of implementing organoid transplantation into humans [18]. Already some new synthetic hydrogel matrices have been developed, however, these are not yet used for multiple tissue types [20, 29].

#### Neoadjuvant treatment

Rising numbers of tumors are being treated with neoadjuvant treatment, resulting in decreased tumor cells at the time of surgery. This reduced number of tumor cells could, as in other tumor models, impair the yield of tumor-derived organoids [12].

#### Lack of microenvironment

One of the major limitations of current organoid technology is the lack of microenvironment. In vivo microenvironment consists of fluctuating gradients of growth factors and extracellular signals, immune cells, blood vessels, inflammatory responses, stromal niches, etc.

In cancer research, the microenvironment plays an essential role. For example, anti-angiogenic drugs, immunotherapy or other stromal-affecting drugs, cannot be tested in tumor-derived organoids [12]. As such, discrepancies of drug sensitivity in organoids compared to in vivo environments exist [22]. Additionally, stromal and micro-environmental factors can also alter the drug response of tumor cells, further complicating the usage of organoids [22]. No robust or effective standard protocol for microenvironment has been established to date, limiting the replicability of organoid studies.

A possible way to address these issues, is to co-culture stromal and immune cells with the organoids, although this is not widely utilized [22]. Other components of the microenvironment, such as the correct in vivo concentrations of growth factors and signaling gradients, are very difficult to mimic in organoids, further complicating matters. Research for a so-called “next-generation organoid” is already been conducted, trying combinations of vascular networks, structured matrix, bioreactors, etc. [29].

Another possible solution to the lack of microenvironment is using a microfluidic platform [12]. This novel technique, also called organ-on-a-chip, is constructed in a top-down fashion in order to fully mimic the in vivo state. Biochemical signals, extracellular environment, mechanical forces, concentration gradients, etc., are all simulated and adjustable [18, 31]. Furthermore, co-culturing immune cells is possible, expanding the possibilities for drug testing. However, further research and optimization is needed [12, 31].

#### Other limitations

It remains unclear whether organoid culture conditions favor healthy organoids over diseased ones. While healthy organoids are readily grown under niche-like conditions, the efficiency of establishing cancer organoid culture from patients is lower in some studies [32]. The reasons for this remain elusive. In pancreatic cancer, the existence of normal-like (Wnt-dependent) and “true” cancer organoids (Wnt-independent) inside one tumor sample has been demonstrated [13, 32]. This raises a question of how many different cancer clones can organoid technology expand beside normal-like organoids: all or only a subset.

Another unresolved issue is whether organoids actually can capture completely the in vivo intratumoral heterogeneity, with only limited knowledge about this aspect of ﻿tumorigenesis [13, 33]. It is also possible that not all the clones are represented in the biopsy specimen, and therefore cannot be expanded like in the in vivo counterpart.

## Organoids of ovarian or fallopian origin

Organoids of ovarian cancer could prove very useful in clinical practice. Since EOC is known as the “silent killer”, a robust therapeutic and diagnostic platform should prove of great significance. Possible future applications of organoids of EOC will be discussed further.

### Organoids of fallopian tubes

It is generally accepted that high-grade serous ovarian carcinoma (HGSOC) originates from serous tubal intraepithelial carcinoma (STIC), growing from the fallopian tube fimbriae [34, 35]. This makes the fallopian tube an interesting tissue to explore EOC carcinogenesis.

Kessler et al. proved that a single fallopian epithelial stem cell had the potential to form differentiated organoids with a great resemblance to the in vivo tissue [36]. These organoids could remain in culture for a long time (> 16 months at the time of publication) and showed a very stable phenotype. In this paper, the existence of fallopian tube stem cells was first proven. The process is mainly dependent on the Wnt and Notch pathways. Especially Notch is of importance since it promotes a secretory cell phenotype, modulated by its signal strength, and regulates the stemness of these cells. Xie et al. confirmed the importance of both the Wnt and Notch pathways in the long-term growth of fallopian tube organoids [37].

Yucer et al. successfully produced fallopian tube organoids from iPSC [34]. This experiment confirmed the importance of the canonical Wnt pathway, as was already proposed by Kessler et al. [36]. The early fallopian tube epithelium formed spheroids, but for the formation of organoids, phenol red - a weak estrogen - proved to be essential. This indicates the importance of estrogen in the differentiation and maturation of the fallopian tubes. Sustaining full grown organoids required both estrogen and progesterone.

In HGSOC, 22% of cases showed an alteration in the Notch pathway leading to a higher proliferation rate [38]. The importance of the Notch pathway in the formation of secretory cell phenotypes, is shown in the previous findings of Chen et al. who proposed serous EOC originates from secretory cell outgrowth [39]. Fallopian tube organoids could thus provide us with an insight in the pathogenesis of EOC – especially HGSOC – by altering the Notch pathway and subsequently inducing secretory cell outgrowth.

### Organoids of ovarian surface epithelial cells

Kwong et al. were the first to develop organoids from normal, healthy human ovarian surface epithelial cells [21]. They demonstrated the role of tumor necrosis factor alpha (TNF-α) in the tumorigenesis of EOC and focused on the function of epithelial inclusion cysts. These inclusion cysts are thought to be a normal physiological postovulatory phenomenon, but previous findings suggest irregular morphology could be a dysplastic precursor of EOC [21]. The irregular inclusion cysts, found in familial high-risk patients, showed loss of basement membranes, loss of epithelial polarity, overexpression of Ki-67, more production of tumor markers and other carcinogenic changes. When TNF-α was added to healthy organoids, the same dysplastic features were induced, suggesting chronic inflammation could lead to the generation of EOC. TNF-α did not induce these changes in monolayer cell cultures, indicating the potential of organoids over traditional cell cultures. However, it remains inconclusive whether this group established a true organoid culture, as no niche growth factors were added into the culture medium and subsequent data is unavailable.

King et al. described organoids from normal mice ovarian surface epithelium and the influence of insulin and insulin-like growth factor 1 [40]. The addition of one of these factors resulted in a hyperplastic ovarian surface epithelium with altered collagen IV deposition. These changes could potentially lead to the development of EOC, especially HGSOC. Organoids proved useful as 2D cultures could not be used for examining the directional growth of this epithelium.

### Organoids of EOC

Until very recently, few publications could be found about organoids of EOC. However, in a recent article by Kopper et al., a major breakthrough in EOC organoids was published [6]. A robust protocol has been presented, capable of cultivating long-term organoids of all main subtypes of EOC. The tissues were harvested from primary tumors or metastatic lesions, including ascites and pleural punctures. These organoids faithfully maintained the genomic landscape, histological aspect and tumor heterogeneity of the original tumor.

Another recent publication by Phan et al. demonstrated a successful method of cultivating organoids derived from two HGSOC patients and one ovarian carcinosarcoma patient [7]. High-throughput drug screening was achievable within 1 week from harvesting the original tumor. Tumor heterogeneity was preserved, and multiple drugs could be tested during the same period. This could be a first step in predicting therapeutic response in EOC prior of starting with systemic treatment in the patient.

A third publication by Maru et al. showed a successful propagation of five ovarian organoids cultures, derived from HGSOC, mucinous and endometrioid ovarian carcinoma [8]. Even borderline ovarian tumor could be used. These organoids were long-term cultured, however exact duration of the cultivation is not specified. Intratumoral heterogeneity was maintained, in addition to a faithful representation of the original histological and genetic features with retention of the somatic mutations found in the original tissue. This group also established a successful drug sensitivity assay using spheroids derived from the ovarian organoids.

These three publications prove that organoids of EOC could be successfully cultivated, indicating a major step in EOC research.

Other publications published data on organoids that were much more short-lived or disclosed less information. Jabs et al. harvested organoids for drug testing from 2D cell cultures of HGSOC [2]. However, these organoids were only grown up to ten days. Also, Hill et al. published short-lived (2 weeks) HGSOC organoids for drug testing, also maintaining their parent heterogeneity and did not develop novel mutations [4]. Another abstract by Hill et al., mentioned short term HGSOC-derived organoids (7–10 days) [10]. This group used organoids to assess DNA damage repair defects and its influence on immune-oncologic agents, apparently providing a possible tool to predict patient response to therapy, however detailed information is lacking. Swan et al. discussed a high-throughput drug screening tool for EOC using organoids from two patients [5]. However, no details about the exact tissue type of these organoids were disclosed. Finally, Gotimer et al. established a short-term organoid platform derived from HGSOC, though very limited data about duration and organoid technique are mentioned [9].

### Summary

Until very recently, no robust method for organoids of ovarian or fallopian tube tissues was published. The three recent publications by Kopper et al., Phan et al., and Maru et al. appear to be a major breakthrough in the research for EOC organoids [6–8]. Further research is needed and using these organoids for diagnostic insights or biobanking could be a next step forward. We acknowledge the limitations of the current technique, but as mentioned before, some efforts have already been made to address these issues in other tissues. However, in ovarian organoids, first a more robust and reliable protocol should be established. The experiences of groups working with other tumor and tissue types could be of great significance, allowing for a more rapid development of EOC-derived organoids.

## Future clinical applications of organoids from EOC

**Fig. 4:**
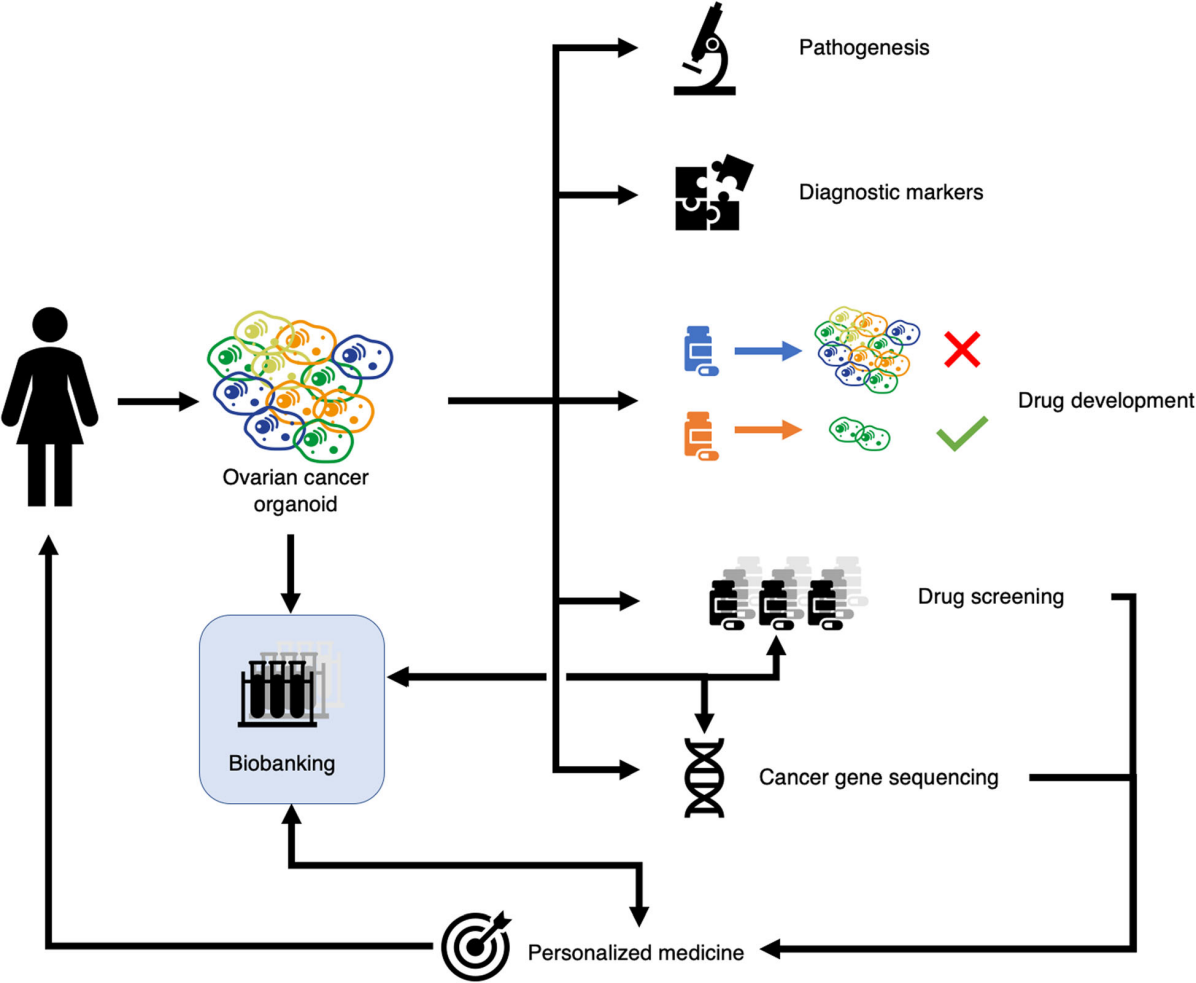
Overview of possible future clinical applications of organoids from epithelial ovarian cancer

Although organoids of EOC are still in an early phase, we investigated the possibilities of this promising technique. We schematically represent these applications in Fig. 4.

### Insights into pathogenesis

Some groups were able to provide valuable insight into the pathogenesis of EOC using organoids. Especially the role of fallopian tube epithelium and ovarian surface epithelial cells are yet to be fully understood in the development of EOC. HGSOC is believed to originate from the fallopian tube, which makes this difficult-to-chart structure an interesting subject to study pathogenesis [41]. Furthermore, organoids prove an exceptionally useful platform, since certain functions and changes in organoids could not be found in monolayer cell cultures [6, 21, 28, 36, 40].

### Diagnostic markers

At present, there are no sensitive or specific markers that can be used in the diagnosis or screening of EOC [41]. A specific and sensitive diagnostic marker would prove useful in screening, diagnosis, and follow-up of patients. There is a large potential to be found in organoids since its immortal structure allows for massive biochemical analysis of the produced proteins. If a large analysis of these proteins could be performed, maybe a better diagnostic marker could be discovered. A nearly infinite number of proteins could be tested in organoids, as these could produce the needed number of samples.

### Drug screening and development

As already shown in other cancer types (such as lymphoma, colorectal cancer, hepatocellular carcinoma, prostate cancer, etc.) and recently in EOC, organoids could potentially be used as a platform for drug testing [7, 28]. With organoids, the expensive and cumbersome processes during the pre-clinical phase of drug development could be streamlined. The main advantages of organoids are the clonality, possibility for high-throughput screening and reduced costs in comparison with animal models or xenografts [28]. Furthermore, rodent models do not always have clinical relevance when used in humans [28]. CRISPR/Cas9 allows for precise genetic manipulation in organoids, which makes drug testing for individual genes more reliable and specific [24]. The TUMOROID trial showed a positive correlation between drug response in organoids and the clinical response of patients with metastatic breast, colorectal and non-small cell lung cancers, which is a first proof-of-concept [12, 22].

However, there are some possible disadvantages. Especially the lack of a tumor microenvironment with blood vessels and immune cells could impair with the tested drugs. Since organoids are still in an early phase, it remains expensive in comparison with traditional 2D cell cultures [28].

### Personalized medicine

Personalized medicine with precise therapy is believed to be the holy grail in cancer treatment, allowing for effective treatment with minimal failure of therapy. Organoids could be used for drug screening in a patient, or more precise drug testing after cancer gene sequencing and linking these results to mutation-based drug sensitivity [12]. This would allow the clinician to select the optimal therapy for the individual patient.

Because the majority of advanced EOC relapses, developing a personalized and effective therapy in these recurrences could prove very useful and potentially improve patient survival. Biobanking would allow for quick-access secondary-line therapy testing. Organoids are already been examined to select optimal therapy in metastatic setting for certain cancers [12].

## Conclusions

Organoids of ovarian and fallopian tissue could offer an insight into the pathogenesis of EOC. Furthermore, EOC organoids could provide an excellent preclinical in vitro platform for drug testing and screening, the discovery of diagnostic markers and eventually be used as a highly personal in vitro model of an individual patient. More progress is needed before this tool could have a clinical purpose, but it could lead to a new epoch for treating patients with EOC. Three recent publications could prove to be an accelerator in EOC organoid research. However, organoids have limitations, primarily due to their lacking microenvironment.

## Supplementary information

**Additional file 1: Table S1.** Search terms concerning reviews of organoids. **Table S2.** Search terms concerning organoids of epithelial ovarian tissue or fallopian tissue. **Table S3.** Articles about organoids of epithelial ovarian or fallopian tissue.

## Data Availability

Not applicable.
