# Supplementary material for: Organoids of epithelial ovarian cancer as an emerging preclinical in vitro tool: a review
Source: J Ovarian Res. 2019 Nov 8;12:105. doi: 10.1186/s13048-019-0577-2 (PMC6839218; doi:10.1186/s13048-019-0577-2)
Supplement: Supplementary file 1 — Additional file 1: Table S1. Search terms concerning reviews of organoids. Table S2. Search terms concerning organoids of epithelial ovarian tissue or fallopian tissue. Table S3. Articles about organoids of epithelial ovarian or fallopian tissue. [file 13048_2019_577_MOESM1_ESM.docx]

Additional Table 3: Articles about organoids of epithelial ovarian or fallopian tissue

| Gotimer K, Chen H, Leiserowitz GS, Smith LH. Short-term organoid culture for drug sensitivity testing in high-grade serous ovarian cancer. Gynecol Oncol. 2019 Jun;153(3):e13. |
| --- |
| Hill SJ, Decker B, Roberts EA, Horowitz NS, Muto MG, Worley MJ, et al. Prediction of DNA repair inhibitor response in short-term patient-derived ovarian cancer organoids. Cancer Discov. 2018;8(11):1404–21. |
| Hill SJ, Lizotte P, Horowitz NS, et al. Abstract 368A: Functional assessment of DNA damage repair defects and the anti-tumor immune response in high grade serous ovarian cancers using patient-derived organoids. Cancer Res. 2019;79(13 Supplement):368A. |
| Jabs J, Zickgraf FM, Park J, Wagner S, Jiang X, Jechow K, et al. Screening drug effects in patient‐derived cancer cells links organoid responses to genome alterations. Mol Syst Biol. 2017 Nov;13(11):955. |
| Kessler M, Fotopoulou C, Winsauer C, Thieck O, Meyer TF. Abstract 3373: Identification of the stem cells in the epithelium of human Fallopian tube. Cancer Res. 2012 Apr;72(8 Supplement):3373–3373. |
| Kessler M, Hoffmann K, Brinkmann V, Thieck O, Jackisch S, Toelle B, et al. The Notch and Wnt pathways regulate stemness and differentiation in human fallopian tube organoids. Nat Commun. 2015;6(1):8989. |
| King SM, Modi DA, Eddie SL, Burdette JE. Insulin and insulin-like growth factor signaling increases proliferation and hyperplasia of the ovarian surface epithelium and decreases follicular integrity through upregulation of the PI3-kinase pathway. J Ovarian Res. 2013 Feb 7;6(1):12. |
| Kopper O, de Witte CJ, Lõhmussaar K, Valle-Inclan JE, Hami N, Kester L, et al. An organoid platform for ovarian cancer captures intra- and interpatient heterogeneity. Nat Med. 2019 Apr 22. |
| Kwong J, Chan FL, Wong K, Birrer MJ, Archibald KM, Balkwill FR, et al. Inflammatory Cytokine Tumor Necrosis Factor α Confers Precancerous Phenotype in an Organoid Model of Normal Human Ovarian Surface Epithelial Cells. Neoplasia. 2009 Jun;11(6):529–41. |
| Maru Y, Tanaka N, Itami M, Hippo Y. Efficient use of patient-derived organoids as a preclinical model for gynecologic tumors. Gynecol Oncol. 2019 Jul;154(1):189–98. |
| Phan N, Hong JJ, Tofig B, Mapua M, Elashoff D, Moatamed NA, et al. A simple high-throughput approach identifies actionable drug sensitivities in patient-derived tumor organoids. Commun Biol. 2019 Dec 25;2(1):78. |
| Phan N, Huang J, Eisenberg D, Memarzadeh S, Soragni A. Abstract 5782: A novel method for high-throughput drug screening in 3D tumor organoids. Cancer Res. 2017 Jul 1;77(13 Supplement):5782–5782. |
| Swan HA, Rosati R, Bridgwater C, Churchill MJ, Watt RM, Shaw RC, et al. Abstract 1619: Personalized medicine: A CLIA-certified high-throughput drug screening platform for ovarian cancer. Cancer Res. 2018 Jul 1;78(13 Supplement):1619–1619. |
| Vogel TJ, Qu Y, Suzuki E, Drapkin R, Sareen D, Svendsen C, et al. Co-culture of human induced pluripotent stem cells (iPSCs) with human fallopian tube epithelium (FTE) induces Pax8 and CK7 expression: Initial steps in modeling fallopian tube epithelium to study serous carcinogenesis. Gynecol Oncol. 2015 Apr;137:206. |
| Xie Y, Park ES, Xiang D, Li Z. Long-term organoid culture reveals enrichment of organoid-forming epithelial cells in the fimbrial portion of mouse fallopian tube. Stem Cell Res. 2018;32(August):51–60. |
| Yucer N, Holzapfel M, Jenkins Vogel T, Lenaeus L, Ornelas L, Laury A, et al. Directed Differentiation of Human Induced Pluripotent Stem Cells into Fallopian Tube Epithelium. Sci Rep. 2017 Dec 6;7(1):10741. |
